# Incidental thyroid lesions detected by FDG-PET/CT: prevalence and risk of thyroid cancer

**DOI:** 10.1186/1477-7819-7-63

**Published:** 2009-08-10

**Authors:** Ja Seong Bae, Byung Joo Chae, Woo Chan Park, Jeong Soo Kim, Sung Hoon Kim, Sang Seol Jung, Byung Joo Song

**Affiliations:** 1 Department of Surgery, The Catholic University of Korea, Seoul, Korea; 2 Department of Nuclear medicine, The Catholic University of Korea, Seoul, Korea

## Abstract

**Background:**

Incidentally found thyroid lesions are frequently detected in patients undergoing FDG-PET/CT. The aim of this study was to investigate the prevalence of incidentally found thyroid lesions in patients undergoing FDG-PET/CT and determine the risk for thyroid cancer.

**Methods:**

FDG-PET/CT was performed on 3,379 patients for evaluation of suspected or known cancer or cancer screening without any history of thyroid cancer between November 2003 and December 2005. Medical records related to the FDG-PET/CT findings including maximum SUV(SUV_max_) and pattern of FDG uptake, US findings, FNA, histopathology received by operation were reviewed retrospectively.

**Results:**

Two hundred eighty five patients (8.4%) were identified to have FDG uptake on FDG-PET/CT. 99 patients with focal or diffuse FDG uptake underwent further evaluation. The cancer risk of incidentally found thyroid lesions on FDG-PET/CT was 23.2% (22/99) and the cancer risks associated with focal and diffuse FDG uptake were 30.9% and 6.4%. There was a significant difference in the SUV_max _between the benign and malignant nodules (3.35 ± 1.69 vs. 6.64 ± 4.12; P < 0.001). There was a significant correlation between the SUV_max _and the size of the cancer.

**Conclusion:**

The results of this study suggest that incidentally found thyroid lesions by FDG-PET/CT, especially a focal FDG uptake and a high SUV, have a high risk of thyroid malignancy. Further diagnostic work-up is needed in these cases.

## Background

Incidentalomas of the thyroid are defined as thyroid lesions identified by radiological imaging, such as ultrasonography (US), computed tomography (CT) and magnetic resonance imaging (MRI) for nonthyroid disease [1,2]. In an autopsy series, the prevalence of thyroid nodules was approximately 50% [3]. Despite the high prevalence of nodules, the annual incidence of palpable thyroid nodules is estimated to be 0.1% in North America [4]. Therefore, most thyroid nodules are identified incidentally than by palpation. While a number of incidentally found thyroid nodules have been identified, the risk of thyroid cancer in these nodules is not well known.

Positron emission tomography (PET)/CT using ^18^F-fluorodeoxyglucose (FDG) is increasingly performed for staging or localization of metastatic disease in patients with various kinds of malignancies. The uptake of the FDG in the normal thyroid gland is homogenous and of low intensity; the normal thyroid gland is usually not visualized on a FDG-PET. [5,6] Focal or diffuse FDG uptake in the thyroid is often seen as an incidental finding. Some studies have reported that the incidence of thyroid incidentalomas with increased FDG uptake is 1.2% – 2.3% on PET examinations [5,7-9]. The risk of malignancy in these studies ranged from 26.7% to 50%.

The recently developed FDG-PET/CT provides the advantages of two modalities; the anatomic information is provided by the spiral CT and the functional information by the FDG-PET. This combined approach has resulted in a significant improvement in both anatomic localization and diagnostic accuracy [10-12]. Since the introduction of the FDG-PET/CT, the vast majority of systems are now produced as combined FDG-PET/CT rather than FDG-PET.

The purpose of this study was to evaluate our institutional experience with incidentally identified thyroid lesions by FDG-PET/CT in suspected or known cancer patients as well as in patients undergoing health screening, and to determine the risk of thyroid malignancy in these patients.

## Methods

### Patients

From November 2003 to December 2005, 3,416 patients underwent FDG-PET/CT in Kangnam St. Mary's Hospital, Seoul, Korea. Among them, 37 patients who were studied because of thyroid cancer were excluded from the study population. Thus, 3,379 patients were included in this analysis. FDG-PET/CT was performed on 666 patients without a previous history of cancer for cancer screening and 2,713 patients received scanning for suspected or known nonthyroid cancer.

### PET/CT Method

**Figure 1:**
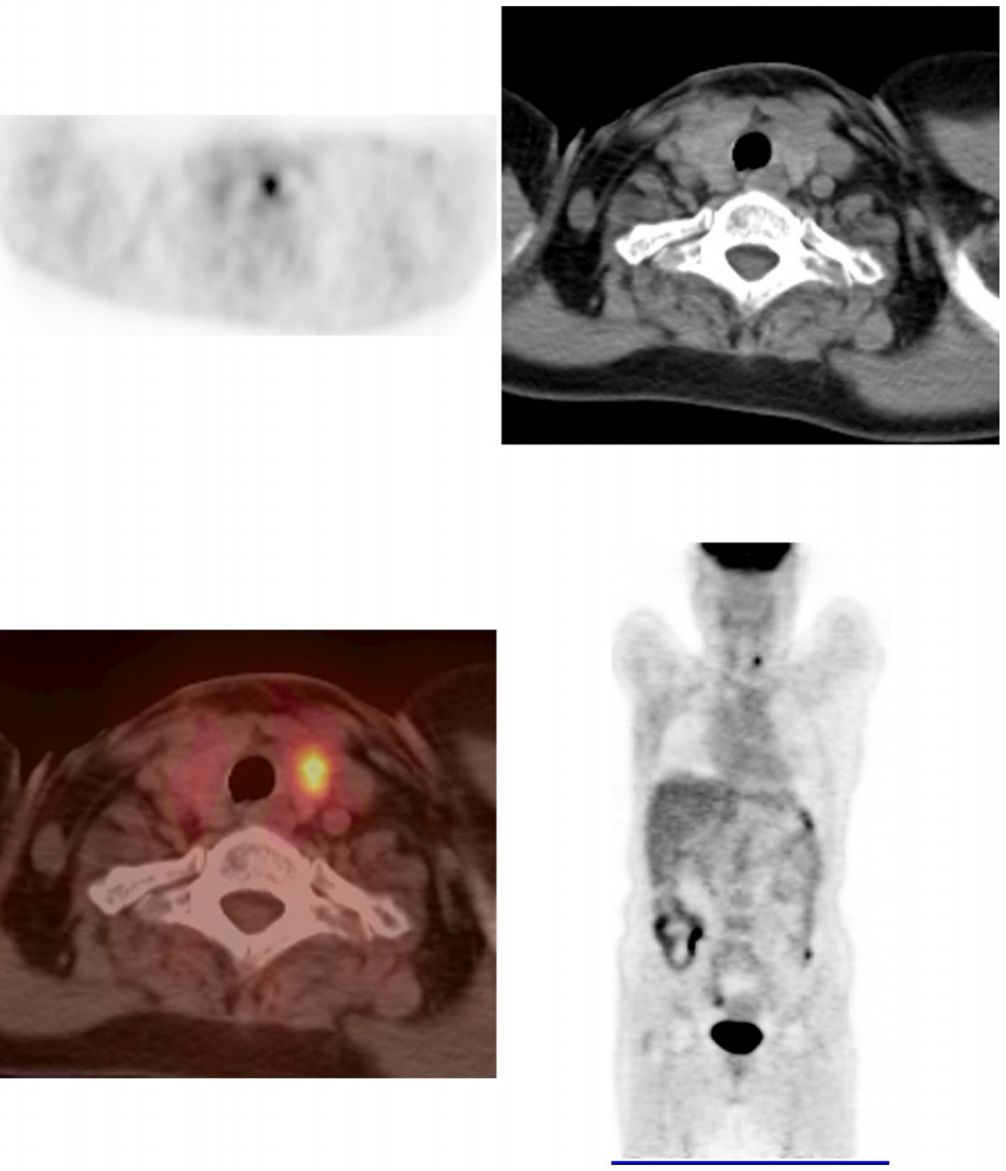
**Focal FDG uptake on PET/CT**. 54 year old female with breast cancer. The ^18^F-FDG PET/CT revealed focal uptake with SUV of 7.6. The patients was performed total thyroidectomy with a final diagnosis of papillary thyroid carcinoma.

**Figure 2:**
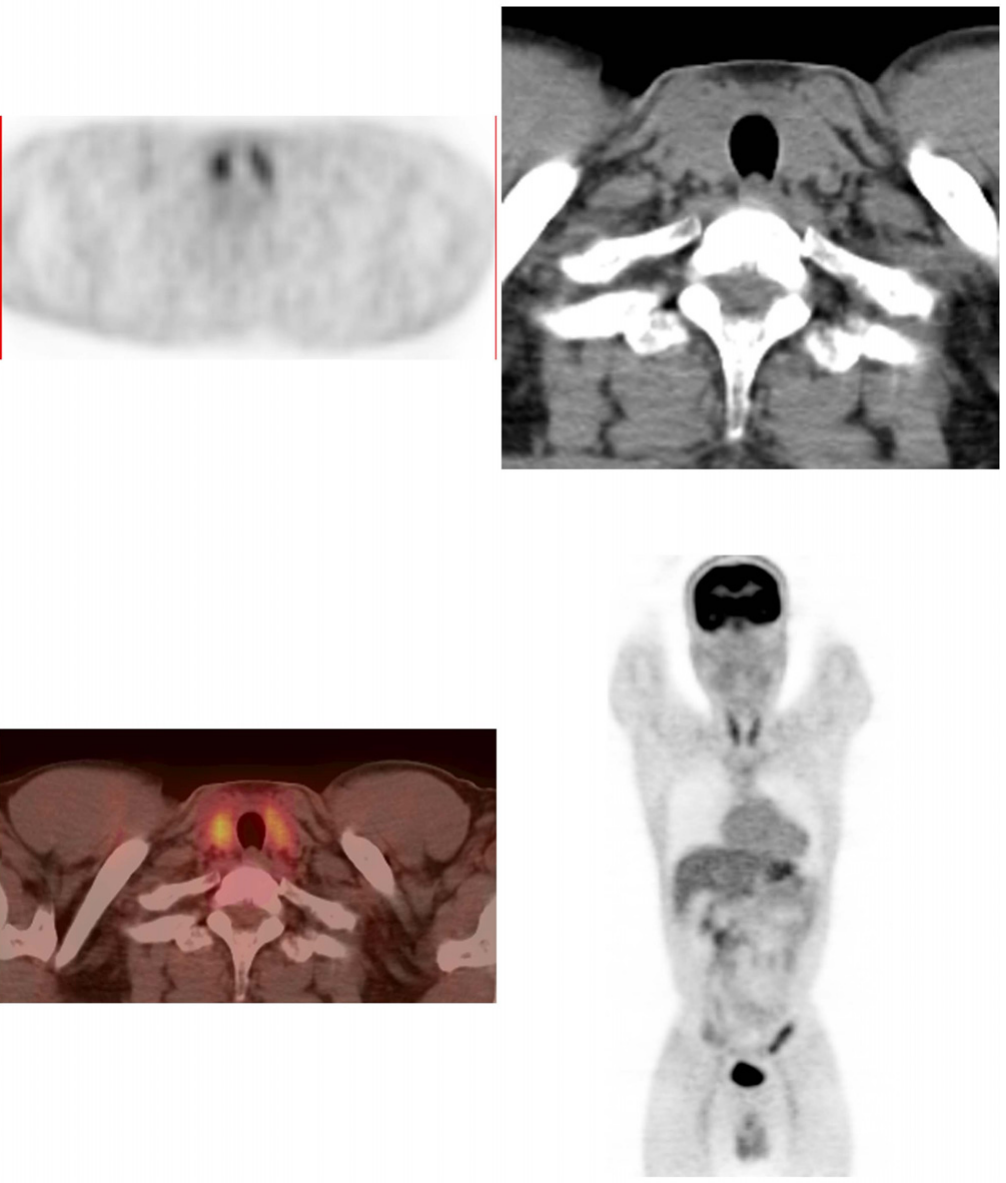
**Diffuse FDG uptatake on PET/CT**. 53 year old male. The ^18^F-FDG PET/CT revealed diffuse uptake with SUV of 3.8. The sonographic features of the thyroid gland were strongly suggestive of the presence of thyroiditis.

For the FDG PET/CT examination, Biograph LSO (Siemens Medical Solutions; Knoxville, TN) integrated with a dual-section helical CT scanner (Somatom Emotion; Siemens) was used. All subjects fasted for at least 6 hours (blood glucose level < 130 mg/dL) before their FDG PET/CT examinations. Image acquisition for the whole body scan started about 60 min after the intravenous administration of 550 MBq of F-18 FDG. Whole body emission scans consisted of 7–8 bed positions for 2 min at each position. In this study, a focal thyroid lesion was defined as a focally increased ^18^F-FDG uptake on the PET images or focal a lesion on the CT images (Fig. 1). A diffuse thyroid lesion was defined as ^18^F-FDG uptake in the whole thyroid gland (Fig. 2). The maximum SUV (SUV_max_) values were obtained for each patient with abnormal thyroid uptake on the FDG-PET/CT. Two experienced nuclear physicians reviewed the images retrospectively.

### Diagnosis and Management of Incidentally Found Thyroid Lesions on PET/CT

Experienced radiologists, who were aware of the FDG-PET/CT findings before US, performed high-resolution ultrasonography (US). US features associated with benign thyroid lesions are purely cystic nodules, hyperechoic nodules, sharp margination, coarse calcification and peripheral vascularity [13]. US features associated with malignancy are microcalcifications, hypoechoic nodules, irregular margins, tall than wide shape and central vascularization [13,14]. A fine needle aspiration (FNA) was performed in patients with abnormal findings on ultrasonography using a 21-gauge needle on a 20-mL syringe under US guidance. A cytology diagnosis was made by experienced cytopathologists. A total thyroidectomy was performed in patients with a malignant neoplasm diagnosed by the FNA cytology. Patients with indeterminate nodules on the FNA cytology underwent frozen biopsy sampling during surgery; the type of operation was dependent on the results of the frozen biopsy.

### Statistics

The statistical analysis was performed using the SPSS (SPSS, Inc., Chicago, IL, USA) software package. A Chi-square test was used to determine the prevalence difference of incidentally found thyroid lesions on the FDG-PET/CT according to gender. A 95% confidence interval was calculated. An independent T-test and the Mann-Whitney U test were performed to compare benign thyroid lesions and malignant thyroid lesions. A receiver-operating-characteristic (ROC) curve analysis was done to differentiate benign from malignant lesions. Spearman's rank correlation was used to assess the relationship between the SUV_max _and the diameter of the thyroid cancer. P values < 0.05 were considered statistically significant. Numeric data were expressed as mean ± standard deviation (SD).

## Results

Three thousand three hundred seventy nine patients (1,484 men, 1,895 women) underwent FDG-PET/CT. In 2,713 patients undergoing FDG-PET/CT for known or suspected cancer, the primary site of malignancy was 893 suffered from breast cancer, 428 from head and neck cancers excluding thyroid cancer, 311 from gastrointestinal cancers, 306 from lung cancer, 297 from gynecologic cancers, 163 from lymphomas, 159 from hepatobiliary cancers, 132 from others, and 24 from metastases of unknown primary origin (Table 1). Of 3,379 patients undergoing FDG-PET/CT, 285 (8.4%) patients were identified as having incidentally found thyroid lesions on the FDG-PET/CT. One hundred thirty-three (3.9%) patients had focal thyroid uptake and 152 (4.5%) patients had diffuse thyroid uptake. Of 285 patients, there were 64 men and 221 women. The prevalence of incidentally found thyroid lesions on FDG-PET/CT was higher in women than in men (11.7% vs. 4.3%; odds ratio = 2.9, 95% CI 2.2–3.9, p < 0.0001). The prevalence of incidentally found thyroid lesions, on FDG-PET/CT, in patients being screened for cancer (58/666; 8.7%) was similar to that of patients with suspected or known cancer (227/2,713; 8.4%).

**Table 1:** Location of primary lesion in patients with incidentally found thyroid lesions on ^18^F-FDG PET/CT.

Primary lesion	Primary diagnosis (n = 3,379)	FDG uptake on PET/CT (n = 285)	Malignancy (n = 23)
Breast	893	97	7
Gastrointestinal	311	25	4
Gynecologic	297	31	3
Lung	306	21	2
Lymphoma	163	19	1
Hepatobiliary	159	8	1
Head and Neck	428	17	0
Others	132	6	0
Unknown malignancy	24	3	0
Cancer screening	666	58	5

FDG; fluorodeoxyglucose (FDG)

PET/CT; Positron emission tomography (PET)/Computed tomography (CT)

### Patients with focal thyroid uptake on FDG-PET/CT

Among 133 patients with incidental focal thyroid uptake on the FDG-PET/CT, 68 (51.1%) patients underwent thyroid US. Among 68 patients, 49 (72%) patients underwent US-guided FNA. Sixty-five patients did not undergo thyroid US because of patient refusal, loss to clinical follow-up or advanced stage of the underlying primary malignancy. Nineteen patients who did not undergo FNA had benign findings on US. Of 19 patients, 3 patients become lost to follow up, 15 patients also had benign findings at follow-up US. One patient die of cervical cancer. The FNA results were as follows: benign in 25 patients (51%), malignant in 17 patients (34.7%), and indeterminate in seven patients (14.3%). Sixteen patients with a cytological diagnosis of a malignant neoplasm underwent operative intervention, and the postoperative pathology diagnosis confirmed the preoperative diagnosis. One patient with a cytological diagnosis of a malignant neoplasm was lost to follow-up. Of 7 patients with cytological diagnosis of an indeterminate nodule, 5 patients underwent operative intervention, one patient did not undergo follow up US and one patient become lost to follow up. Among 5 patients who underwent operative intervention, papillary carcinomas were found in three patients, follicular carcinoma in one patient and nodular hyperplasia in one patient.

### Patients with diffuse thyroid uptake on FDG-PET/CT

Among 152 patients with diffuse thyroid uptake on the FDG-PET/CT, 31 (20.4%) patients underwent thyroid US. US findings showed thyroiditis or diffuse goiter in 14 (45.2%) patients, a benign-looking nodule in 10 (32.2%) patients, an indeterminate nodule in four (12.9%) patients, and normal findings in three (9.7%) patients. A FNA was performed in four patients with an indeterminate nodule on US. Benign lesions were found in two patients. A malignant neoplasm and an indeterminate lesion were found in the remaining two. Two patients with a cytological diagnosis of a malignant neoplasm or an indeterminate nodule underwent operative intervention. A papillary cancer was found in one patient and a follicular carcinoma was found in the other patient.

### Characteristics of patients with a malignancy on the FNA or pathology

**Figure 3:**
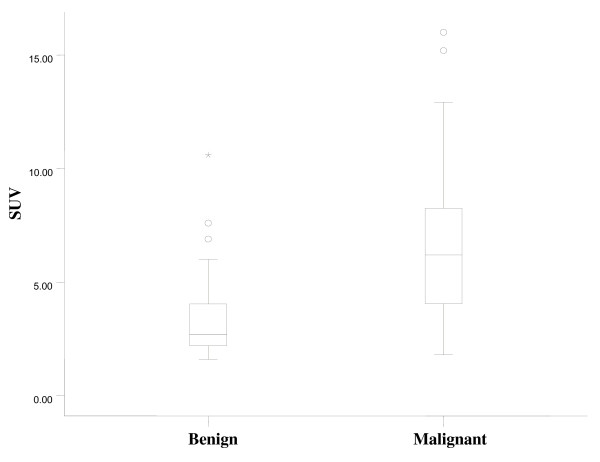
**SUV_max _of benign and malignant thyroid lesions on PET/CT**. Side by side box plots of SUV_max _by groups. Statistically significant differences was found in SUV_max _between benign lesions and malignant lesions (P < 0.001).

**Figure 4:**
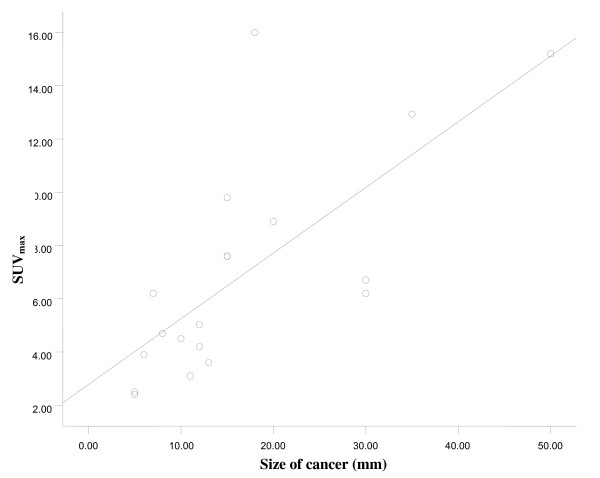
**Correlation between SUV_max _and cancer size**. Spearman r test was performed to find correlation between SUV_max _and thyroid cancer size. (Spearman r = 0.776, 95% confidence interval 0.50–0.91, P = 0.0001).

**Figure 5:**
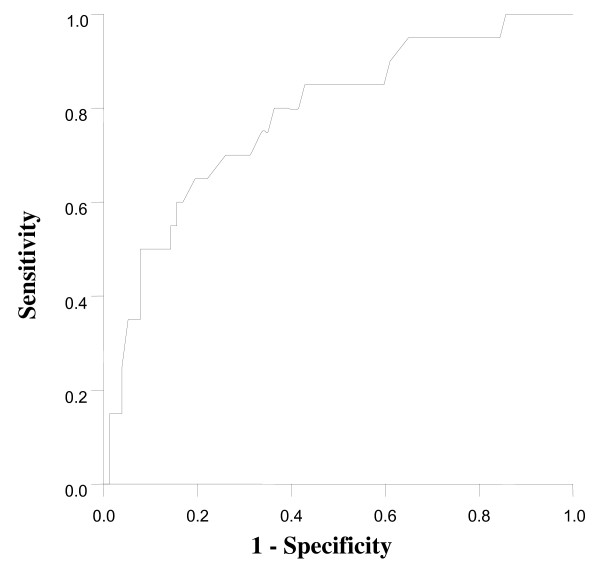
**ROC curve to differentiate between benign and malignant lesions on PET/CT**.

In 23 patients, the diagnosis of a malignancy was made by histopathology. The malignancies were papillary carcinomas in 21 patients and follicular carcinomas in two patients. The patients included 17 women and 6 men with a mean age 53.5 ± 11.0 (range 47–72) years and 52.9 ± 10.8 (range 34–67) years, respectively. Twenty-two patients underwent a total thyroidectomy. One patient was lost to follow up. Focal uptake was significantly associated with a higher prevalence of cancer when compared to patients with diffuse uptake (P = 0.009) (Table 2). The average value of SUV_max _of malignant thyroid lesions was significantly higher than that of benign thyroid lesions. (Fig. 3) There was no correlation between the SUV_max _and the diameter of the benign lesion (Spearman r = 0.179, 95% -0.15 – 0.47, P = 0.271) There was a significant correlation between the SUV_max _and the diameter of the cancer (Spearman r = 0.776, 95% CI 0.50–0.91, P = 0.0001) (Fig. 4). When the ROC curve and SUV_max _value for differentiating benign from malignant lesions were used, the cut-off value for the SUV_max _was 3.5 from the ROC curve based on the present study. The sensitivity was 80.0% and the specificity was 66.1% (Fig. 5)

**Table 2:** Comparison between benign and malignant thyroid lesions incidentally detected on ^18^F-FDG PET/CT.*

	Benign (n = 76)	Malignant (n = 23)	P value
Age	54.24 ± 9.79	52.73 ± 10.31	0.527
Sex			
Male	12	6	0.354
Female	64	17	
Size by US†	1.81 ± 1.08	1.54 ± 0.90	0.289
Mean SUV_max_.	3.35	6.64	<0.001
Pattern of FDG uptake			
Focal FDG uptake	47	21	0.009
Diffuse FDG uptake	29	2	

US; Ultrasonography

SUVmax; Maximum standardized uptake value

FDG; fluorodeoxyglucose (FDG)

* 99 patients underwent further diagnostic confirmation

## Discussion

Our result showed that incidentally found thyroid lesions including diffuse or focal FDG uptake on the FDG-PET/CT occurred with a prevalence of 8.4% (285/3379), which is higher than reported previously using FDG-PET [5,15]. The prevalence of focal FDG uptake in the thyroid gland was also high in comparison with previous reports [5,8,9,15]. The use of the FDG-PET/CT combination may contribute to identifying thyroid uptake. Diffuse FDG uptake in the thyroid gland is associated with benign conditions such as thyroiditis, nodular goiter and Graves' disease [16-18]. In the present study, most ultrasound examinations of patients with diffuse FDG uptake in thyroid gland showed benign disease such as thyroiditis and multinodular goiter.

Incidentally found thyroid lesions have become an important part of clinical practice. Many patients are incidentally discovered during radiological imaging studies for nonthyroidal disease or as part of a health screening program. Ultrasound is the most sensitive diagnostic modality for differentiating benign from malignant lesions. There are no specific findings to suspect malignancy on CT and MRI unless there is invasion into adjacent structures. Even though FDG-PET/CT is gradually being used more frequently for cancer staging, localization and surveillance of known cancers or cancer screening, there are no definitive findings for the diagnosis of thyroid cancer. In our study, the cancer prevalence of patients with incidentally found thyroid lesions on FDG-PET/CT was 23.2% (23/99), which was lower than reported in previous studies [1,5,9]. The cancer prevalence with focal uptake on the FDG-PET/CT was 30.9% (21/68), still lower than previous studies. However, that risk for a malignancy, in those patients with focal uptake on the FDG-PET/CT, is high and additional work up is needed in these patients. Some authors have suggested that the SUV_max_on the FDG-PET/CT might be useful to differentiate benign from malignant thyroid lesions [8,9,19]. Kang et al [8] reported that the average SUV_max_of malignant lesions (16.5 ± 4.7) was significantly higher than that of benign lesions (6.5 ± 3.8), whereas others reported that there was no difference in the SUV_max _between benign and malignant lesions [5,15]. Our study showed that malignant thyroid lesions had a significantly higher SUV_max _than benign thyroid lesions. The pattern of uptake in the thyroid gland on FDG-PET/CT was noted to be useful in differentiating the patients with a malignancy. Focal uptake on the FDG-PET/CT significantly correlated with an increased risk of malignancy in comparison with a diffuse uptake. These findings are similar to the results documented by other reports [8,15].

The advantages of the FDG-PET/CT over FDG-PET include anatomic localization of focal uptake and evaluation of CT characteristics of the thyroid lesions detected on the FDG-PET/CT. Choi et al [1] reported improved accuracy for characterizing thyroid nodules on the FDG-PET/CT using CT attenuation. Yi et al [20] also reported that four malignant nodules had low attenuation on CT images. However, CT could not definitively discriminate a benign from a malignant nodule. The gold standard for diagnosing a benign versus a malignant thyroid nodule is ultrasonography and fine needle aspiration biopsy. The CT can help detect a focal thyroid nodule in patients with or without mild FDG uptake.

In this study, the prevalence of incidentally found thyroid lesions in suspected or known cancer patients on the FDG-PET/CT was similar to that of patients receiving scanning for health screening. Furthermore, the prevalence of histologically proven thyroid malignancy was not different between these two groups. Our results are not consistent with a previous report [1]. Our results suggest that the prevalence of incidentally found thyroid lesions is similar in patients with known cancer and in the general population. In addition, the primary location of a known or suspected cancer showed no significant difference of the prevalence of incidentally found thyroid lesions on the FDG-PET/CT.

The FDG-PET/CT has no a clear role in the preoperative evaluation for differentiated thyroid cancer patients. Mitchell et al [19] enrolled 31 patients with thyroid nodules and demonstrated that the sensitivity and specificity of FDG-PET/CT were 60% and 91%. The positive predictive value and negative predictive value of the FDG-PET/CT was 75% and 83%. Jeong HS et al [21] showed that the FDG-PET/CT did not provide any additional benefit over either the US or the contrast-enhanced CT for cervical lymph node metastases in patients with papillary thyroid cancer, because of the relatively low levels of glucose metabolism. Our study showed that the sensitivity and specificity of the FDG-PET/CT were 80.0% and 66.1%, respectively, and that the FDG-PET/CT did not provide information, preoperatively, on cervical lymph node metastases in three patients with postoperatively proven cervical lymph node metastasis of thyroid cancer. The clinical significance of preoperative FDG-PET/CT for differentiated thyroid cancer requires further investigation.

The main limitation of this study was the retrospective analysis. Forty percent of patients who had incidentally identified focal uptake by thyroid lesions on the FDG-PET/CT did not have further evaluation. In the majority of these patients, the extent of the primary disease did not allow for a meaningful investigation of the incidentally found thyroid lesions by FDG-PET/CT. Another limitation was the small number of patients with incidentally found thyroid lesions on the FDG-PET/CT and histologically proven thyroid nodules. Additional investigation with a larger patient sample and a prospective study design are needed for further study of this issue.

In patients with incidentally found thyroid lesions on FDG-PET/CT, the prevalence of malignancy is 23.2%. The prevalence in cancer screening subjects and in patients with suspected and known cancer was similar. The factors that were related with an increased risk of a malignancy were focal FDG uptake on the FDG-PET/CT and a high SUV_max_. The presence of risk factors such as a focal FDG uptake and a high SUV_max. _on the FDG-PET/CT warrant ultrasonography and fine needle aspiration biopsy.

## Competing interests

The authors declare that they have no competing interests.

## Authors' contributions

JSB drafted the manuscript and contributed to conception and design. BJC contributed to acquisition and analysis of data. WCP, JSK and SSJ participated in the design of the study and revised ir critically for important intellectual content. SHK participated in the design of study and performed the statistical analysis. BJS conceived of the study and pariticipated in its design and coordination. All authors read and approved the final manuscript.
